# NF-kB in Signaling Patterns and Its Temporal Dynamics Encode/Decode Human Diseases

**DOI:** 10.3390/life12122012

**Published:** 2022-12-02

**Authors:** Sanaa Almowallad, Leena S. Alqahtani, Mohammad Mobashir

**Affiliations:** 1 Department of Biochemistry, Faculty of Sciences, University of Tabuk, Tabuk 71491, Saudi Arabia; 2 Department of Biochemistry, College of Science, University of Jeddah, Jeddah 23445, Saudi Arabia; 3 SciLifeLab, Department of Oncology and Pathology, Karolinska Institutet, P.O. Box 1031, S-17121 Stockholm, Sweden; 4 Department of Biosciences, Faculty of Natural Science, Jamia Millia Islamia, New Delhi 110025, India; 5 Special Infectious Agents Unit—BSL3, King Fahd Medical Research Center, King Abdulaziz University, Jeddah 21362, Saudi Arabia

**Keywords:** NF-κB signaling, signaling dynamics, cell-fate decision, encoding and decoding, cellular information, human diseases

## Abstract

Defects in signaling pathways are the root cause of many disorders. These malformations come in a wide variety of types, and their causes are also very diverse. Some of these flaws can be brought on by pathogenic organisms and viruses, many of which can obstruct signaling processes. Other illnesses are linked to malfunctions in the way that cell signaling pathways work. When thinking about how errors in signaling pathways might cause disease, the idea of signalosome remodeling is helpful. The signalosome may be conveniently divided into two types of defects: phenotypic remodeling and genotypic remodeling. The majority of significant illnesses that affect people, including high blood pressure, heart disease, diabetes, and many types of mental illness, appear to be caused by minute phenotypic changes in signaling pathways. Such phenotypic remodeling modifies cell behavior and subverts normal cellular processes, resulting in illness. There has not been much progress in creating efficient therapies since it has been challenging to definitively confirm this connection between signalosome remodeling and illness. The considerable redundancy included into cell signaling systems presents several potential for developing novel treatments for various disease conditions. One of the most important pathways, NF-κB, controls several aspects of innate and adaptive immune responses, is a key modulator of inflammatory reactions, and has been widely studied both from experimental and theoretical perspectives. NF-κB contributes to the control of inflammasomes and stimulates the expression of a number of pro-inflammatory genes, including those that produce cytokines and chemokines. Additionally, NF-κB is essential for controlling innate immune cells and inflammatory T cells’ survival, activation, and differentiation. As a result, aberrant NF-κB activation plays a role in the pathogenesis of several inflammatory illnesses. The activation and function of NF-κB in relation to inflammatory illnesses was covered here, and the advancement of treatment approaches based on NF-κB inhibition will be highlighted. This review presents the temporal behavior of NF-κB and its potential relevance in different human diseases which will be helpful not only for theoretical but also for experimental perspectives.

## 1. Introduction

The idea that structure reflects function is a recurring one in biology. The genome is arguably the most well-known example of a biological structure that foretells physiological activity. One can determine whether coding DNA encodes a protein domain, binding site, conserved motif, or hairpin structure by understanding the sequence structure of the DNA [1,2,3,4,5]. These illustrations show that the structural elements of a cell contain functional information that is encoded. If we could measure cellular structures in enough detail, one may claim that they contain all the necessary information. Is this the only possible method for encoding biological information or is it possible to learn about the features of biological activity that cannot be learned by studying static structures alone? Here, the main concern is about the recent development in cell biology that provides a different way for information to be sent in cells such as by the motion of signaling molecules. As per the nomenclature, dynamics refers to the form of the curve that shows how a molecule’s concentration, activity, modification state, or localization alters over time (temporal dynamics) [6,7,8,9,10]. This kind of signaling stores data in the temporal signal’s frequency, amplitude, duration, or other characteristics. As a result, it is richer and more complicated than communicating through the state of a signaling molecule instantly. We have focused on the comprehensive overview of what is known about the dynamics of many biological systems, concentrating on systems that have been well investigated and have undergone analysis using a variety of quantitative measurement and perturbation techniques. Through these illustrations, we are able to draw broad conclusions regarding the function of dynamics in biology and the potential benefits of transferring information via the dynamics of signaling molecules [11,12,13,14,15,16,17,18,19].

Since Sen and Baltimore’s discovery of nuclear factor kappa B (NF-κB) in 1986, a great deal of work has been put forward to clarify the roles that this transcription factor plays in the body. NF-κB is a member of the Rel-homology-domain family of transcription factors. The transactivation of numerous target genes involved in immunity, inflammation, and proliferation mostly depends on its component p65/RelA. Its activity is strictly controlled by the B protein inhibitors (IBs) and the B kinase proteins (IKKs), and as a result, growth factors, cytokines, and cell proliferation are expressed. The main functions of NF-κB are to trigger immunological and inflammatory reactions as well as to control apoptosis [20,21,22,23,24]. Its targets include the genes that make cytokines, chemokines, and anti-apoptotic substances as well as cell adhesion molecules. By hiding its RHD, the inhibitory (inhibitor B (IB)) molecules (such as IκBα) confine NF-κB to the cytoplasm. A second complex known as IkappaB kinase (IKK) is activated when cytokines such as interleukin-1 (IL-1) or tumor necrosis factor (TNF) excite a cell [25,26].

The cytosolic IκB kinase activity was initially linked to a massive protein complex of 700–900 kDa that was capable of precisely phosphorylating IκBα on serines 32 and 36. The regulatory subunit IKKγ (NEMO) and two catalytically active kinases, IKKα and IKKβ, were found in this complex after it was purified. IKKα and IKKβ are ubiquitously expressed proteins with leucine zippers, carboxy-terminal helix–loop–helix domains, and amino-terminal kinase domains (HLH). The kinases dimerize through the leucine zipper, and mutations in this region leave the kinases inactive. In contrast, the HLH is not required for dimerization but is crucial for optimal kinase activity. The connection between the regulatory subunit IKKα and IKKβ, which is regulated by a hexapeptide sequence (LDWSWL) on IKKs known as the NEMO binding domain (NBD), depends on the carboxy-terminal parts of IKKα and IKKβ. The phosphorylation of two serines in the sequence motif SLCTS of the T-loop sections in at least one of the IκB kinases is required for the activation of the IKK complex [27].

IB is made ready for proteolysis by ubiquitin by being phosphorylated by the active IKK complex. Following this, NF-κB moves into the nucleus to start the transcription of the target genes. Due to a lack of linkages to specific human disorders, NF-κB signaling has not received much attention in the human setting despite being involved in essential cellular processes. Nevertheless, a number of publications over the last year have identified faulty NF-κB function in a number of hereditary illnesses, including ectodermal dysplasia (ED), familial expansile osteolysis (FEO), primary lymphedema (PL), and incontinentia pigmenti (IP). In this study, we have discussed the precise abnormalities in the NF-κB pathway as well as the genetic mutations that result from these four illnesses [20,28].

**Figure 1:**
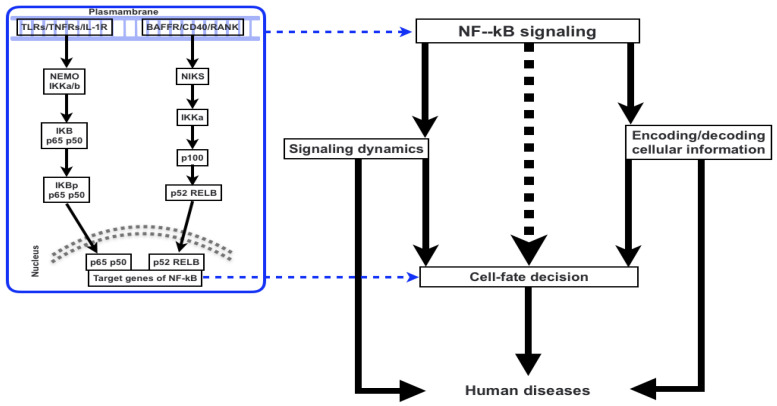
An overview of the work presented in this review.

Since abnormalities in NF-κB function influence a variety of physiological systems and organs in mice, including the immune system, fetal liver, skin, limbs, and the osteoclast lineage, so we have discussed broad-spectrum role for it. The etiology of ED, FEO, PL, and IP has been explained in part by our understanding of the NF-κB pathway, but the phenotypes of human diseases have also shed light on the route itself. An old protein transcription factor known as NF-κB is thought to control innate immunity. By connecting pathogenic signals and cellular danger signals, the NF-κB signaling pathway organizes cellular defenses against encroaching pathogens (Figure 1). In reality, several studies have demonstrated that NF-κB functions as a network hub for intricate biological signaling [29,30,31]. To this purpose, it has been proposed that NF-κB is a master regulator of metabolic cascades that have been preserved throughout evolution.

Physiologist Claud Bernard proposed in 1854 that complex systems such as the human body require the ability to self-regulate their internal environment in order to live. Almost 60 years later, Walter B. Cannon used the term “homeostasis” to describe this essential characteristic of living things. Homeostasis, despite its origin, is a very dynamic process in which only a small number of internal physicochemical parameters are kept within a specific range, or are homeostatically managed. Homeostatic regulation is crucial at the system, tissue, and cellular levels in complex organisms. For instance, the blood’s pH is regulated by the brain, lungs, kidneys, and red blood cells; adult stem cells renew tissues to preserve their integrity; and cells keep the baseline concentrations of Ca^2+^, Na^+^, and K^+^ within certain ranges [31,32,33]. Homeostatic regulation depends on biochemical networks known as cell signaling, which provide cells with the capacity to detect physicochemical stimuli, analyze information, and carry out the best biological reactions. Here, we focus on the new conceptual and methodological developments that are improving our knowledge of the molecular processes underlying homeostatic regulation [7,34,35].

Progenitors are the ancestors of all cells. Cellular variety and specialization can be established throughout development through the differentiation of progenitor cells. Additionally, in recent years, we know that the majority of tissues reserve undifferentiated progenitors that probably contribute to the tissue’s homeostasis during the course of its lifespan. These cells’ differentiation during homeostasis is probably a tightly controlled process. Progenitor growth and differentiation may become dysregulated in illness, but this is not well understood. For instance, the cancer stem cell theory contends that populations of tumor cells that resemble healthy adult stem cells biologically are the source of malignant tumors [36,37,38]. These might be endogenous stem cells that have developed somatic mutations over time that lead to cancer. Another illustration is fibrosis, which is characterized by an overgrowth of myofibroblasts that produce components of the extracellular matrix that render tissues non-compliant. The origins of myofibroblasts in fibrotic tissues are poorly known, but it has long been assumed that these cells come from nearby regions. Thus, cell fate decisions in development and disease could be of potential interest and this could update on the processes that regulate cell destiny differentiation, techniques for studying and quantifying cell fate differentiation, and evaluations of the current understanding of how cell fate differentiation goes haywire in illness or disease models. Cells can transmit and receive information by managing the temporal behavior (dynamics) of their signaling molecules, according to an increasing number of studies. We go through what is known about the dynamics of various signaling networks and how they affect cellular responses in this review.

We discuss the emerging general principles in the field, paying particular attention to how the type and amount of stimulus are represented in temporal patterns, how signaling dynamics affect cellular outcomes, and how particular dynamical patterns are both generated and interpreted by the organization of molecular networks. In order to conclude, we also discussed about probable functions for signaling molecules’ dynamics in transferring cellular information and prospective applications for disease therapy. To achieve our goal for this review study, most relevant review and research works as well as the recent work were studied and have also cited them in the required places.

## 2. NF-κB Signaling

Controlling inflammation is one of the main functions of NF-κB proteins, which suggests that they focus on the body’s intricate defensive systems when inflammation is present [28,39,40,41]. This is accomplished by controlling the expression of several crucial genes involved in the crucial process, including chemokines and pro-inflammatory cytokines, in both a positive and negative manner. IL-1, for example, and tumor necrosis factor alpha (TNF-α) are powerful inducers of NF-κB. Additionally, NF-κB aids reduce inflammation, which subsequently impairs NF-κB activity [42,43,44]. The anti-inflammatory, pro-autophagy, and anti-insulin resistance protein Sirtuin 1 is less abundant as people age and become obese because NF-κB is produced more frequently. By attaching to the promoter region of the microRNA miR-34a, which prevents the formation of nicotinamide adenine dinucleotides (NAD), NF-κB raises its levels in the body, causing Sirtuin 1 levels to drop. Senescence-associated secretory phenotype (SASP) factors are produced by senescent cells as a result of a positive feedback loop between NF-κB and IL-1α. NF-κB and CD38, an enzyme that breaks down nicotinamide adenine dinucleotides, also mutually induce one another [45,46,47,48].

With this review, we want to better understand how NF-κB activation affects mitochondrial function. It is expected that the reader is already familiar with the fundamentals of mitochondrial biology. RelA, RelB, c-Rel, p100, and p150 are the five different proteins that make up the NF-κB transcription factor family. For dimerization, DNA binding, and interaction with l-B inhibitors, they have RHD, which are crucial. The v-rel oncogene from the T-strain of the reticuloendotheliosis virus (REV), which produces the embryonic lymphatic tumor, is the source of the domain’s name due to their same sequence. Additionally, the transcription activation domain is present in RelA, RelB, and c-Rel (transcription activation domain (TAD)) [44,49,50,51,52]. Additionally, TADs are missing from p100 and p105, which are precursor proteins for NF-kB, which, following proteolysis, creates the p50 and p52 subunits. As a result, NF-κB is a general term that can refer to a family of dimer proteins made by various substances. Additionally, they all contain the RelA, RelB, c-Rel, p50, and p52 subunits. The majority of cells express the NF-κB dimer p50/RelA, for which many research works have been performed in the past. P100 is processed to create p52 by the two serine residues (in the phosphorylation areas) and the lysine residues (in the ubiquitination region). Ankyrin (ANK) repeats: ANK IkB dysregulation has been known to include serine residues (phosphorylation sites) and lysine residues (ubiquitination sites).

The NF-κB is activated by the two signaling pathways, canonical/classical and non-canonical/alternative (Figure 1). The canonical/classical pathway is activated by extracellular stimuli such as TNF-alpha, RANK (receptor activator of nuclear factor kappa B), TCR (T-cell receptor), CD30, CD40, and LPS (bacterial lipopolysaccharides). These extracellular stimuli change the IKK trimetric complex, which is made up of two catalytic subunits of IKKα and IKKβ and a regulatory subunit of IKKγ (also known as NF-κB essential modulator or NEMO) leads to the phosphorylation of IKB inhibitor (IKB-α) at different sites (i.e., Ser 32 and Ser 36) as a result of the continued ubiquitination of IKBα [53,54]. The p50/p65-containing free NF-kB dimer is activated, translocates to the nucleus, and binds to the promoter region of responsive genes to trigger their transcription. The B-cell activator receptor (BAFF-R), lymphotoxin beta receptor (LTBR), CD40 activating NIK kinase phosphorylating the IKK complex containing IKB dimerized complex, further activating the RelB/p100 to RelB/p52, and transcription are just a few extracellular stimuli that can activate the non-canonical/alternative pathway [28,44,55].

The ability to receive and interpret information from both the intracellular and external environments, initiate and carry out biological reactions, and interact with one another is created through cell signaling. Cell signaling is ultimately in charge of preserving homeostasis at the cellular, tissue, and systemic levels. In order to understand how cells coordinate the transitions between states under developing and adult organisms in healthy and pathological settings, cell signaling is the focus of considerable study efforts. With an emphasis on how single-cell analytical tools uncover processes driving cell-to-cell variability, signaling plasticity, and collective cellular responses, we summarized current knowledge of how cell signaling functions at various spatial and temporal scales in this article [8,56,57,58,59,60].

Eukaryotic cells frequently employ NF-κB as a regulator of genes that govern cell survival and proliferation. As a result, NF-κB has been misregulated in a wide variety of human tumor types, making it constitutively active. Inactive NF-κB prevents the production of genes that would otherwise lead the cell to undergo apoptosis and maintain cell proliferation. Cancer is characterized by mutations or the abnormal expression of proteins that regulate NF-κB signaling, which impair the ability of the malignant cell to coordinate with the rest of the body. This is demonstrated by metastasis as well as by the immune system’s ineffective removal of the tumor. When a normal cell is removed from the tissue to which it belongs or when its genome cannot function in harmony with tissue function, it might die. These events rely on the feedback control of NF-κB, which is defective in cancer. Increased apoptosis sensitivity and subsequent cell death are caused by NF-κB defects [29,61,62,63,64]. This is due to the fact that NF-κB controls genes that prevent apoptosis, particularly TRAF1 and TRAF2, and as a result, suppresses the activities of the caspase family of enzymes, which are essential to the majority of apoptotic processes. As in 41% of nasopharyngeal carcinoma, colorectal cancer, prostate cancer, and pancreatic tumors, tumor cells have increased NF-κB activity. This is caused by mutations in the genes that code for the NF-κB transcription factors or in the genes that regulate NF-κB activity (such the IB genes); in addition, certain tumor cells produce substances that activate NF-κB. By inhibiting NF-κB, tumor cells may cease growing, perish, or develop increased sensitivity to the effects of anti-tumor medications. As a result, pharmaceutical companies are actively researching NF-κB as a target for anti-cancer treatment [65,66,67,68,69].

The development of antitumor therapy based on the suppression of NF-κB activity is justified by convincing experimental data that identify NF-κB as a key promoter of tumorigenesis; however, care should be taken when considering anti-NF-κB activity as a general therapeutic strategy in the treatment of cancer because data also show that NF-κB activity increases tumor cell sensitivity to apoptosis and senescence. Furthermore, it has been demonstrated that the alternative NF-κB is a Fas transcription repressor and that conventional NF-κB is a Fas transcription activator. Because NF-κB encourages Fas-mediated apoptosis in cancer cells, inhibiting NF-κB may restrict Fas-mediated apoptosis and reduce the ability of host immune cells to prevent tumor growth [70,71,72,73,74]. Here, we have explained the role of NF-κB in different human diseases, which covers a wide range of NF-κB activation mechanisms and potentially associated SMs [1,75,76,77].

## 3. Signaling Dynamics and the Association with Signaling Parameters

**Figure 2:**
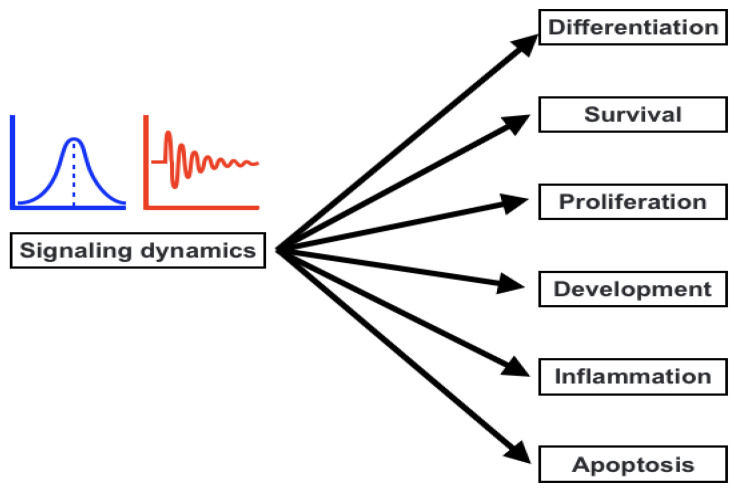
Signaling dynamics controlling various cellular decisions.

Information must be transferred from a receptor to the nucleus through a process called signal transduction. This procedure is essential for regulating cellular activity and destiny. Apoptosis, proliferation, and differentiation are influenced by the dynamics of signaling activation and inhibition (Figure 2). Therefore, it is crucial to comprehend the variables that affect both transient and persistent reaction. Using a mathematical method, we investigated the variables that can change how downstream signaling molecules are activated in order to answer this issue. Loops (feedforward and feedback loops), crosstalk of signal transduction pathways, and changes in the concentration of signaling molecules are the aspects that we looked into. According to our findings, the feedback loop and cross-talks that directly block the target protein dominate in regulating the temporary cellular response [75,76,77]. The ability to receive and interpret information from both the intracellular and external environments, initiate and carry out biological reactions, and interact with one another is created through cell signaling. Cell signaling is ultimately in charge of preserving homeostasis at the cellular, tissue, and systemic levels. In order to understand how the cell coordinates the transitions between states under developing and adult organisms in healthy and pathological settings, cell signaling is the focus of considerable study efforts. With an emphasis on how single-cell analytical tools uncover processes driving cell-to-cell variability, signaling plasticity, and collective cellular responses, we summarize current knowledge of how cell signaling functions at various spatial and temporal scales in this article [28,78,79,80,81]. Cell signaling therefore coordinates the transition between cellular states with the molecular machinery (such as ERBB-dependent cytoskeletal rearrangements or DNA damage repair) in response to a stimulus in order to maintain cell homeostasis and function. Similar to this, signaling affects cellular choices that control the development of multicellular animals and the homeostasis of adult tissues by combining cell-autonomous and non-cell-autonomous pathways. We are able to deterministically explain each step of these mechanisms, but it is becoming more and more clear that stochastic models are better able to capture the behavior of cells within a population. For instance, stem cells that, while proliferating, demonstrate a specific likelihood to either differentiate or self-renew, maintain the homeostasis of skin and the esophageal epithelium. To ensure tissue regeneration, this likelihood is precisely calibrated. However, the disruption of cell signaling by cell-autonomous pathways or cell-to-cell communication can change this equilibrium, resulting in illnesses such as cancer [82,83,84,85,86].

In recent decades, phospholipid-based signal sensing has seen significant advancements. Numerous studies have shown that the homeostasis of phospholipids is maintained by an intricate and dynamic network of metabolic processes that are controlled by a number of enzymes, whose activities are extremely sensitive to external and intracellular stimuli. A sizable and structurally complex collection of signaling molecules is formed as a result of the multiplicity of phospholipid metabolic pathways. Even within a single cell, the sheer number of effectors leads to the development of a highly intricate network of signaling pathways that follow the activation of phospholipid signaling. Recent studies have uncovered crucial facets of phospholipid signaling, the relevance of their spatiotemporally diverse and dynamic molecular profile, and their multiple locations in various extracellular fluids, as well as specific membrane microdomains, distinct intramembrane pools, and various subcellular sites and compartments. Phospholipids, along with the metabolic products they produce, are essential for signaling events that are involved in a variety of biological processes, including those that control survival, growth, differentiation, shape, motility, activation, and death. Recent evidence that numerous phospholipids and their derivatives play a crucial role as transcriptional regulators of complicated nuclear signaling pathways revealed an intriguing feature of phospholipid signaling. In fact, certain phospholipid species have the ability to operate as non-membrane associated lipids to precisely bind to and functionally control the activation of particular nuclear receptors. In addition, several nuclear receptors have the ability to attach phospholipid head groups to essential phospholipid signaling enzymes, which can subsequently change the phospholipid head group with special kinetic features. Thus, phospholipid signaling is a network of metabolic pathways that is intricately regulated and crucial for the management of homeostasis, intercellular communication, and efficient cellular responses [87,88,89,90].

Recent research also demonstrated that the role of phospholipid signaling in the control of extracellular vesicles added yet another layer of intricacy to its already intricate processes. These tiny, secreted vesicular structures, which play a role in intercellular communication and the preservation of physiological homeostasis, are becoming a new frontier in signal transduction. These vesicles seem to carry particular phospholipid enzymes or signals, offering a method of efficiently transporting them across long distances to various cells without dilution or destruction. Given the importance of phospholipid signaling and the variety of cell regulatory functions it performs, it should come as no surprise that its dysregulation is the root of many diseases. The onset of a number of human diseases is driven by defects in phospholipid signaling, according to recently accumulated evidence. Numerous studies have shown that changes in phospholipid signaling can accelerate the development of a number of illnesses, including cancer, cardiovascular, and neurological problems, as well as developmental and degenerative diseases. Understanding the triggers that cause the signaling transition to a pathogenic function and developing innovative treatment techniques are two pertinent, ongoing research areas [80,91,92,93].

It has been demonstrated in earlier research that the temporal dynamics may be related to signaling factors such as signal molecule concentration, feedback/feedforward (positive/negative), crosstalk (positive/negative) between the pathways, and reaction rate [18,75,76,94,95]. These analyses into many parameters have been conducted for various signaling pathways, including MAPK. The strength of the receptor and input signals could perhaps have an impact on the nature of the temporal response of the downstream signaling molecules, according to the prior research. Similarly to this, while the rate of reaction (kinetic parameters) can simply increase or decrease the response strength, feedback loops and route crosstalk can entirely change the nature of the signaling response [76,77,96,97,98,99,100,101,102].

## 4. Cell-Fate Decision

**Figure 3:**
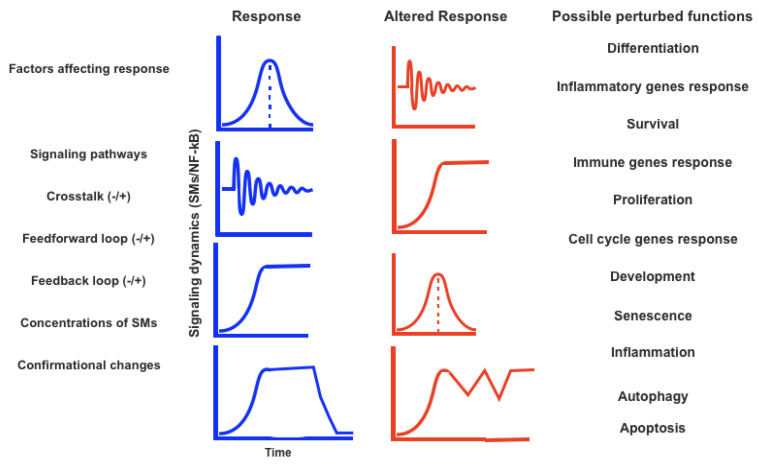
Signaling dynamics are known to control various cellular/cell-fate decisions. Signaling dynamics are associated with specific responses (downstream SMs). Targeted perturbations reveal the role of dynamics in cellular responses.

Understanding the mechanisms that control cell fate decisions is a core objective of developmental and stem cell biology. The contributions of alterations in transcriptional programming, epigenetic modifications, and biochemical differentiation signals are covered in the majority of studies on the regulation of cell destiny decisions. Recent research has discovered that the regulation of cell destiny decisions is also significantly influenced by other facets of cell biology. Intracellular molecular regulatory networks and external environmental factors interact intricately to control the destiny of cells. We are able to analyze the molecular specifics of these regulatory mechanisms in ever-greater depth because of recent improvements in experimental technology. These cues serve both upstream and downstream of developmental signaling pathways and can play either a permissive or instructional role. They are a part of a wider network of signaling (Figure 1 and Figure 2) and for detailed relevance, Figure 3 has been shown which infers the aberration to cell-fate decision. Regulation of cell destiny has a direct impact on human health and tissue homeostasis. Research on cell fate choice identifies important regulators, aids in comprehending the mechanisms, and offers fresh ideas for treating clinical disorders associated with aberrant cell development [21,22,103,104,105,106,107,108,109].

A specialized organelle for protein folding and trafficking, the endoplasmic reticulum (ER) is extremely sensitive to changes in intracellular homeostasis and external stimuli. Misfolded proteins build up in the ER as a result of changes in the protein-folding environment, which has a significant impact on a number of cellular signaling processes, such as energy generation, inflammation, differentiation, and death. The unfolded protein response (UPR) is a group of adaptive signaling pathways that have developed to address protein misfolding and re-establish a favorable environment for protein folding. Recent developments: The UPR and ER stress have both been connected to the production of reactive oxygen species (ROS). ROS, which can be formed in the cytosol and a number of organelles, including the ER and mitochondria, are important for many cellular activities. According to studies, ER stress, which may in turn trigger the formation of ROS in the ER and mitochondria, can be brought on by a change in the ER’s redox equilibrium. Despite the fact that oxidative stress and ER stress frequently occur in pathologic situations, it is unclear whether or how these stressors interact. Additionally, it is not known how modifications to the ER’s protein-folding environment result in oxidative stress. Additionally, it is uncertain how the creation of ROS and protein misfolding cause apoptosis in cells and contribute to a number of degenerative disorders. In terms of future perspectives, the discovery of innovative therapies for many human diseases will benefit from a deeper knowledge of the underlying processes that maintain the homeostasis and redox state of protein folding [16,110,111,112,113].

Through progenitor cell differentiation, cellular diversity and specialization can be established throughout development. The majority of tissues reserve undifferentiated progenitors, which are likely involved in the tissue’s homeostasis during the duration of its existence, as we have also learnt in recent years. The differentiation of these cells during homeostasis is probably a carefully regulated process [114,115,116]. Although this is not well understood, progenitor development and differentiation may become dysregulated in disease. For instance, the cancer stem cell theory claims that the biological origin of malignant tumors is populations of tumor cells that resemble healthy adult stem cells. These could be endogenous stem cells that, over time, underwent somatic mutations that result in cancer. Another example is fibrosis, which is characterized by an overabundance of myofibroblasts that create extracellular matrix components that make tissues less flexible. Although little is known about the origins of myofibroblasts in fibrotic tissues, it has long been believed that these cells originate from the immediate area [117,118,119,120,121].

## 5. Encoding and Decoding Cellular Information

Cells modulate the dynamics of intracellular signaling molecules (SMs) to transmit and receive information through the signal transduction process. Cellular decision making critically depends on the temporal dynamics of SMs. For instance, prolonged Erk activation following NGF administration causes PC-12 cells to differentiate, whereas transient Erk activation results in proliferation. According to previously published research, a number of serious illnesses seem to be brought on by abnormalities in the signal transduction pathway. The crucial factor is the length or kind of cellular response, which appears to be directly related to the choice of the cell’s fate. The cells go through apoptosis, proliferation, or differentiation depending on the kind of biological response (transient, sustained, or partly adapted) [1,16,122,123,124,125,126]. Understanding how the signaling pathways interact to produce a temporary or long-lasting physiological response is thus a crucial stage in the signal transduction process. A cascade of linked biochemical processes that finally control the elements in charge of cellular phenotypic activities influence the response of a cell to environmental inputs. The signaling apparatus was first envisioned as a combination of separate, linear routes. However, the present perception of the system as a complex network has supplanted the more recent elaboration of the breadth and diversity of intra-pathway crosstalk. These days, it is believed that the cell’s signaling network serves as the primary functional module, which is coupled to a number of additional modules that control phenotypic function. These latter ones include those that control the cell’s secretory, motile, and translational activities. A noteworthy feature of signaling is that transmission and information processing are intertwined. Through intra-cascade feedback control and cross-talk with other channels, the interactions between individual components serve as the interfaces for information computing. The context-specificity of the cellular response is a result of the signaling network’s capacity to process information [85,90,127,128,129,130,131].

Within certain cell types, the kinetics of cell signaling and transcriptional regulatory activity vary in response to the same stimulus. There is a lot of interest in using single-cell-size data in addition to researching network connections to clarify the non-random parts of the variability involved in cellular decision making. Based on an immediate link between the molecular processes, previous studies have taken into account the information flow between the signaling and transcriptional domains. These results suggest a restricted binary on/off encoding technique that undervalues the complexity of biological information processing and, thus, the value of data at the single-cell scale. Here, instead of focusing on chemical abundances, we adopt a unique approach that reframes the information transmission problem as incorporating the dynamic aspects of signaling (Figure 3). We use a computational method to investigate whether and how the patterns of transcriptional regulatory activity might provide insight into the temporal evolution of signaling. This work also summarizes two methodological factors: (1) the dynamic characteristics of signaling that significantly change transcriptional regulatory patterns (encoding); and (2) the temporal history of signaling that can be inferred from snapshots of transcriptional activity taken at the single-cell scale (decoding). In contrast to transcription factor activity patterns, which were instructive of the activation and deactivation kinetics of signaling, immediate early gene expression patterns were indicative of signaling peak retention kinetics. Additionally, the network’s information processing characteristics fluctuated, with each component encoding a particular portion of the dynamic signaling qualities. In order to understand the dynamic multiplexing of the signaling properties at each of these network components, we created unique sensitivity and information transfer maps. Two groups that corresponded with network patterns and could be separated by transcriptional feedforward vs. feedback interactions were discovered by the unsupervised clustering of the maps. By finding the downstream snapshot measurements necessary for deducing particular dynamical aspects of upstream signals important in the control of cellular responses, our novel computational technique has an influence on the single-cell size investigations [9,16,132,133,134,135,136,137].

In order to identify the functional feedback influencing the observed dynamics, it might be useful to examine the dynamics of signaling molecules in response to various stimuli. For instance, a negative feedback between ERK and Son of Sevenless (SOS) in the EGF pathway contributes to the variations in ERK dynamics in response to EGF or NGF. Additionally, NGF signaling, but not EGF signaling, persists after receptor internalization, which helps maintain ERK activation. Positive feedback on ERK activation through PKC is also supported by research. The inference is that changes in the identity and the connection of different route components are what cause the different responses to EGF and NGF, which are mediated by the dynamics of ERK [13,111,138,139,140,141].

## 6. NF-κB Signaling and Molecular Targets for Therapeutic Purpose of Human Diseases

Numerous factors that contribute to human diseases such as different types of cancers include NF-κB. The development of cancer is known to be linked to inflammation, which depends on the reciprocal activation of NF-κB and inflammatory cytokines. The anticancer effects of therapy are diminished by both constitutive and therapeutic-induced NF-κB activation. Understanding the functions of NF-κB in cancer aids the development of methods for cancer therapy and prevention [25,26]. A thorough analysis of NF-κB in each type of disease is essential in this regard due to the complexity of NF-κB involvement in many human diseases. To increase efficacy and lower systemic toxicity, more disease-specific NF-κB inhibiting techniques are sought. NF-κB potentially regulates the transcription process, apoptosis, and proliferation by interacting/affecting with/the critical biological pathways via their components (genes/proteins). These pathway components are targeted for therapeutic purposes. Bcl-2 family members, IAP family members, ROS, p53, MDM2, MDR1, DR5, FASL, Bax, and more are well-known targets for therapeutic purposes. Furthermore, the targeting approach may differ such as the application nanoparticles coated herbal drugs and/or pure herbal drugs [25,26,142,143].

Potential NF-κB blockers include non-steroidal anti-inflammatory drugs (NSAIDs) such as sulindac, aspirin, ibuprofen, indomethacin, and COX-2 inhibitors. These either directly suppress NF-κB at critical junctures along the NF-κB activation pathway or indirectly suppress the inflammatory cell response to do so. It has been thoroughly investigated whether combining these medications with anticancer medicines will promote chemoprevention or chemosensitization. Curcumin (diferuloylmethane), eicosapentaenoic acid (EPA), luteolin, and other naturally occurring anti-inflammatory chemicals are also able to block NF-κB, making them another class of NF-κB-blocking medications for cancer therapy and prevention. These substances obstruct NF-κB at various points along the route. Celestrol blocks NF-DNA B’s binding, apigenin and anacardic acid block IKK, resveratrol blocks p65 phosphorylation, epicatechin blocks p65 translocation to the nucleus, and p65 phosphorylation, respectively. It is important to highlight that these compounds are mostly antioxidants, and their potential role in the prevention of cancer may include controlling the redox state of the cell. The modification of redox, however, might be a factor in NF-κB blocking. It is also known that luteolin inhibits TNF-α-induced NF-κB in lung cancer cells by activating superoxide. Luteolin’s inhibition of NF-κB causes TNF-α-induced cancer cell survival to switch to apoptosis. Luteolin may function as a possible chemopreventive agent due to its capacity to change TNF-α from a tumor promoter to a tumor suppressor due to its ability to block NF-κB. TNF-α is engaged in inflammation-associated carcinogenesis [144,145,146].

## 7. Conclusions and Future Perspectives

When thinking about dynamics’ functional role, the next question that comes to mind is how cells understand various dynamical patterns. What molecular processes, in other words, are required to recognize time-dependent patterns and convert these patterns into distinctive phenotypic responses? Identifying the mechanisms that decode dynamics continues to be one of the field’s most difficult objectives despite the fact that numerous studies have identified functional roles for particular temporal behaviors, and only a small portion of these have precisely determined how different dynamical patterns are distinguished at the molecular level to cause different downstream responses. When addressing the functional relevance of dynamics, the difficult topic is how cells understand various dynamical patterns. Which molecular processes are required to recognize time-dependent patterns and convert them into different phenotypic responses? We have presented details about the NF-κB signaling roles in human diseases, details of signaling dynamics, cell-fate decision, and encoding/decoding of cellular information into final cell-fate decision. We consider it to be of potential interest which could be helpful in not only understanding and exploring the dynamics of different signaling pathway for multiple purposes, including in precise therapeutic approach for different human diseases.

## Data Availability

Not applicable.

## References

[1] Purvis J.E., Lahav G. (2013). Encoding and Decoding Cellular Information through Signaling Dynamics. Cell.

[2] Klil-Drori A.J., Azoulay L., Pollak M.N. (2016). Cancer, obesity, diabetes, and antidiabetic drugs: Is the fog clearing?. Nat. Rev. Clin. Oncol..

[3] Davis D.M., Purvis J.E. (2015). Computational analysis of signaling patterns in single cells. Semin Cell Dev Biol..

[4] Garcia-Bernardo J., Eppstein M.J. (2015). Evolving modular genetic regulatory networks with a recursive, top-down approach. Syst. Synth. Biol..

[5] Batchelor E., Loewer A., Mock C., Lahav G. (2011). Stimulus-dependent dynamics of p53 in single cells. Mol. Syst. Biol..

[6] Regev-Rudzki N., Wilson D.W., Carvalho T.G., Sisquella X., Coleman B.M., Rug M., Bursac D., Angrisano F., Gee M., Hill A.F. (2013). Cell-Cell Communication between Malaria-Infected Red Blood Cells via Exosome-like Vesicles. Cell.

[7] Ruch R.J. (2002). Intercellular communication, homeostasis, and toxicology. Toxicol. Sci..

[8] Cotari J.W., Voisinne G., Dar O.E., Karabacak V., Altan-Bonnet G. (2013). Cell-to-Cell Variability Analysis Dissects the Plasticity of Signaling of Common Chain Cytokines in T Cells. Sci. Signal..

[9] Poltorak M., Arndt B., Kowtharapu B.S., Reddycherla A.V., Witte V., Lindquist J.A., Schraven B., Simeoni L. (2013). TCR activation kinetics and feedback regulation in primary human T cells. Cell Commun. Signal..

[10] Lim W.A., Pawson T. (2010). Phosphotyrosine Signaling: Evolving a New Cellular Communication System. Cell.

[11] Matolcsi M., Giordano N. (2015). A Novel Explanation for Observed CaMKII Dynamics in Dendritic Spines with Added EGTA or BAPTA. Biophys. J..

[12] El-Kafrawy S.A., El-Daly M.M., Bajrai L.H., Alandijany T.A., Faizo A.A., Mobashir M., Ahmed S.S., Ahmed S., Alam S., Jeet R. (2022). Genomic profiling and network-level understanding uncover the potential genes and the pathways in hepatocellular carcinoma. Front. Genet..

[13] Orton R.J., Sturm O.E., Vyshemirsky V., Calder M., Gilbert D.R., Kolch W. (2005). Computational modelling of the receptor-tyrosine-kinase-activated MAPK pathway. Biochem. J..

[14] Hogenesch J.B., Ueda H.R. (2011). Understanding systems-level properties: Timely stories from the study of clocks. Nat. Rev. Genet..

[15] Stelzl U., Worm U., Lalowski M., Haenig C., Brembeck F.H., Goehler H., Stroedicke M., Zenkner M., Schoenherr A., Koeppen S. (2005). A Human Protein-Protein Interaction Network: A Resource for Annotating the Proteome. Cell.

[16] Yao G., Lee T.J., Mori S., Nevins J.R., You L. (2008). A bistable Rb–E2F switch underlies the restriction point. Nat. Cell Biol..

[17] Sobie E.A. (2011). Bistability in Biochemical Signaling Models. Sci. Signal..

[18] Kholodenko B.N. (2006). Cell-signalling dynamics in time and space. Nat. Rev. Mol. Cell Biol..

[19] Schleich K., Lavrik I.N. (2013). Mathematical modeling of apoptosis. Cell Commun. Signal..

[20] Prashar Y., Ritu, Gill N.S. (2017). Emerging role of various signaling pathways in the pathogenesis and therapeutics of atherosclerosis. Rev. Vasc. Med..

[21] Loewer A., Lahav G. (2006). Cellular Conference Call: External Feedback Affects Cell-Fate Decisions. Cell.

[22] Niiro H., Clark E.A. (2002). Decision making in the immune system: Regulation of B-cell fate by antigen-receptor signals. Nat. Rev. Immunol..

[23] White K.L., Rider D.N., Kalli K.R., Knutson K.L., Jarvik G.P., Goode E.L. (2011). Genomics of the NF-κB signaling pathway: Hypothesized role in ovarian cancer. Cancer Causes Control.

[24] Ricaño-Ponce I., Wijmenga C. (2013). Mapping of Immune-Mediated Disease Genes. Annu. Rev. Genom. Hum. Genet..

[25] Habashy N.H., Kodous A.S., Abu-Serie M.M. (2021). Targeting ROS/NF-κB signaling pathway by the seedless black Vitis vinifera polyphenolsin CCl. Sci. Rep..

[26] Abu-Serie M.M., Hamouda A.F., Habashy N.H. (2021). Acacia senegal gumattenuates systemic toxicityin CCl. Sci. Rep..

[27] Oeckinghaus A., Ghosh S. (2009). The NF-kappaB family of transcription factors and its regulation. Cold Spring Harb. Perspect. Biol..

[28] Manning B.D., Cantley L.C. (2007). AKT/PKB Signaling: Navigating Downstream. Cell.

[29] Mostafizar M., Cortes-Pérez C., Snow W., Djordjevic J., Adlimoghaddam A., Albensi B.C. (2021). Challenges with Methods for Detecting and Studying the Transcription Factor Nuclear Factor Kappa B (NF-κB) in the Central Nervous System. Cells.

[30] Yan F., Liu L., Wang Q. (2020). Combinatorial dynamics of protein synthesis time delay and negative feedback loop in NF-κB signalling pathway. IET Syst. Biol..

[31] Prescott J.A., Mitchell J.P., Cook S.J. (2021). Inhibitory feedback control of NF-κB signalling in health and disease. Biochem. J..

[32] ARAUJO R., LIOTTA L. (2006). A control theoretic paradigm for cell signaling networks: A simple complexity for a sensitive robustness. Curr. Opin. Chem. Biol..

[33] Restifo N.P., Smyth M.J., Snyder A. (2016). Acquired resistance to immunotherapy and future challenges. Nat. Rev. Cancer.

[34] Inoki K., Kim J., Guan K.-L. (2012). AMPK and mTOR in Cellular Energy Homeostasis and Drug Targets. Annu. Rev. Pharmacol. Toxicol..

[35] Buszczak M., Signer R.A.J., Morrison S.J. (2014). Cellular Differences in Protein Synthesis Regulate Tissue Homeostasis. Cell.

[36] Furusawa C., Kaneko K. (2012). A Dynamical-Systems View of Stem Cell Biology. Science.

[37] de Souza N. (2014). A systems view of cellular reprogramming. Nat. Meth..

[38] Burzyn D., Kuswanto W., Kolodin D., Shadrach J.L., Cerletti M., Jang Y., Sefik E., Tan T.G., Wagers A.J., Benoist C. (2013). A Special Populationof Regulatory T Cells Potentiates Muscle Repair. Cell.

[39] Gutierrez-Arcelus M., Rich S.S., Raychaudhuri S. (2016). Autoimmune diseases—Connecting risk alleles with molecular traits of the immune system. Nat. Rev. Genet..

[40] Kurosaki T., Shinohara H., Baba Y. (2010). B Cell Signaling and Fate Decision. Annu. Rev. Immunol..

[41] Baxt L.A., Garza-Mayers A.C., Goldberg M.B. (2013). Bacterial Subversion of Host Innate Immune Pathways. Science.

[42] Shalkami A.S., Hassan M., Bakr A.G. (2017). Anti-inflammatory, antioxidant and anti-apoptotic activity of diosmin in acetic acid-induced ulcerative colitis. Hum. Exp. Toxicol..

[43] Bouwmeester T., Bauch A., Ruffner H., Angrand P.-O., Bergamini G., Croughton K., Cruciat C., Eberhard D., Gagneur J., Ghidelli S. (2004). A physical and functional map of the human TNF-α/NF-κB signal transduction pathway. Nat. Cell Biol..

[44] Helmi N., Alammari D., Mobashir M. (2022). Role of potential COVID-19 immune system associated genes and the potential pathways linkage with type-2 diabetes. Combinatorial Chemistry & High Throughput Screening..

[45] Zhou T., Hu Z., Yang S., Sun L., Yu Z., Wang G. (2018). Review Article Role of Adaptive and Innate Immunity in Type 2 Diabetes Mellitus. J. Diabetes Res..

[46] Stephenson E., Reynolds G., Botting R.A., Calero-Nieto F.J., Morgan M.D., Tuong Z.K., Bach K., Sungnak W., Worlock K.B., Yoshida M. (2021). Single-cell multi-omics analysis of the immune response in COVID-19. Nat. Med..

[47] Chalkiadaki A., Guarente L. (2015). The multifaceted functions of sirtuins in cancer. Nat. Rev. Cancer.

[48] Machado-Oliveira G., Ramos C., Marques A.R.A., Vieira O.V. (2020). Cell Senescence, Multiple Organelle Dysfunction and Atherosclerosis. Cells.

[49] Long E.O., Sik Kim H., Liu D., Peterson M.E., Rajagopalan S. (2013). Controlling Natural Killer Cell Responses: Integration of Signals for Activation and Inhibition. Annu. Rev. Immunol..

[50] Kuballa P., Nolte W.M., Castoreno A.B., Xavier R.J. (2012). Autophagy and the Immune System. Annu. Rev. Immunol..

[51] Bezbradica J.S., Medzhitov R. (2009). Integration of cytokine and heterologous receptor signaling pathways. Nat. Immunol..

[52] Tigno-Aranjuez J.T., Bai X., Abbott D.W. (2012). A Discrete Ubiquitin-Mediated Network Regulates the Strength of NOD2 Signaling. Mol. Cell. Biol..

[53] Källstig E., McCabe B.D., Schneider B.L. (2021). The Links between ALS and NF-κB. Int. J. Mol. Sci..

[54] Bagaev A.V., Garaeva A.Y., Lebedeva E.S., Pichugin A.V., Ataullakhanov R.I., Ataullakhanov F.I. (2019). Elevated pre-activation basal level of nuclear NF-κB in native macrophages accelerates Lps- induced translocation of cytosolic NF-κB into the cell nucleus. Sci. Rep..

[55] Hernandez L., Kim M.K., Noonan A.M., Sagher E., Kohlhammer H., Wright G., Lyle L.T., Steeg P.S., Anver M., Bowtell D.D. (2015). A dual role for Caspase8 and NF. Nat. Publ. Group.

[56] Martin G.S. (2003). Cell signaling and cancer. Cancer Cell.

[57] Schlessinger J. (2000). Cell signaling by receptor tyrosine kinases. Cell.

[58] Yang J.-M., Chi W.-Y., Liang J., Takayanagi S., Iglesias P.A., Huang C.-H. (2021). Deciphering cell signaling networks with massively multiplexed biosensor barcoding. Cell.

[59] Tyson J.J., Chen K.C., Novák B. (2003). Sniffers, buzzers, toggles and blinkers: Dynamics of regulatory and signaling pathways in the cell. Current Opinion in Cell Biology.

[60] Danko C.G., Hah N., Luo X., Martins A.L., Core L., Lis J.T., Siepel A., Kraus W.L. (2013). Signaling Pathways Differentially Affect RNA Polymerase II Initiation, Pausing, and Elongation Rate in Cells. Mol. Cell.

[61] Han B., Li X., Ai R.-S., Deng S.-Y., Ye Z.-Q., Deng X., Ma W., Xiao S., Wang J.-Z., Wang L.-M. (2022). Atmospheric particulate matter aggravates cns demyelination through involvement of TLR-4/NF-kB signaling and microglial activation. eLife.

[62] Heltberg M.L., Krishna S., Jensen M.H. (2018). On chaotic dynamics in transcription factors and the associated effects in differential gene regulation. Nat. Commun..

[63] Dorrington M.G., Fraser I.D.C. (2019). NF-κB Signaling in Macrophages: Dynamics, Crosstalk, and Signal Integration. Front. Immunol..

[64] Kuznetsova T., Prange K.H.M., Glass C.K., Winther M.P.J. (2019). Transcriptional and epigeneticregulation of macrophagesin atherosclerosis. Nat. Rev. Cardiol..

[65] Moore K.E., Carlson S.M., Camp N.D., Cheung P., James R.G., Chua K.F., Wolf-Yadlin A., Gozani O. (2013). A General Molecular Affinity Strategy for Global Detection and Proteomic Analysis of Lysine Methylation. Mol. Cell.

[66] Fabbri G., Rasi S., Rossi D., Trifonov V., Khiabanian H., Ma J., Grunn A., Fangazio M., Capello D., Monti S. (2011). Analysis of the chronic lymphocytic leukemia coding genome: Role of NOTCH1mutational activation. J. Exp. Med..

[67] Ma Y., Galluzzi L., Zitvogel L., Kroemer G. (2013). Autophagy and Cellular Immune Responses. Immunity.

[68] Khan N., Mukhtar H. (2013). Biochemical Pharmacology. Biochem. Pharmacol..

[69] Bui J.D., Schreiber R.D. (2007). Cancer immunosurveillance, immunoediting and inflammation: Independent or interdependent processes?. Curr. Opin. Immunol..

[70] Koosha S., Alshawsh M.A., Looi C.Y., Seyedan A., Mohamed Z. (2016). An Association Map on the Effect of Flavonoids on the Signaling Pathways in Colorectal Cancer. Int. J. Med. Sci..

[71] Visvader J.E. (2011). Cells of origin in cancer. Nature.

[72] Gonda T.J., Ramsay R.G. (2015). Directly targeting transcriptional dysregulation in cancer. Nat. Rev. Cancer.

[73] da Silva H.B., Amaral E.P., Nolasco E.L., de Victo N.C., Atique R., Jank C.C., Anschau V., Zerbini L.F., Correa R.G. (2013). Dissecting Major Signaling Pathways throughout the Development of Prostate Cancer. Prostate Cancer.

[74] Feinberg A.P., Koldobskiy M.A., Göndör A. (2016). Epigenetic modulators, modifiers and mediators in cancer aetiology and progression. Nat. Rev. Genet..

[75] Mobashir M., Madhusudhan T., Isermann B., Beyer T., Schraven B. (2014). Negative Interactions and Feedback Regulations Are Required for Transient Cellular Response. Sci. Rep..

[76] Mobashir M., Schraven B., Beyer T. (2012). Simulated evolution of signal transduction networks. PLoS ONE.

[77] Mobashir M. (2013). Mathematical Modeling and Evolution of Signal Transduction Pathways and Networks. Ph.D. Thesis.

[78] Vafai S.B., Mootha V.K. (2013). A Common Pathway for a Rare Disease?. Science.

[79] Pant D.K., Ghosh A. (2006). A systems biology approach for the study of cumulative oncogenes with applications to the MAPK signal transduction pathway. Biophys. Chem..

[80] Rousseau F., Schymkowitz J. (2005). A systems biology perspective on protein structural dynamics and signal transduction. Curr. Opin. Struct. Biol..

[81] Mosaddeghi P., Eslami M., Farahmandnejad M., Akhavein M., Ranjbarfarrokhi R., Khorraminejad-Shirazi M., Shahabinezhad F., Taghipour M., Dorvash M., Sakhteman A. (2020). A systems pharmacology approach to identify the autophagy-inducing effects of Traditional Persian medicinal plants. Sci. Rep..

[82] Werner H.M.J., Mills G.B., Ram P.T. (2014). Cancer Systems Biology: A peek into the future of patient care?. Nat. Rev. Clin. Oncol..

[83] Wang E., Zou J., Zaman N., Beitel L.K., Trifiro M., Paliouras M. (2013). Seminars in Cancer Biology. Semin. Cancer Biol..

[84] Wang E., Zaman N., Mcgee S., Milanese J.-S., Masoudi-Nejad A., O’Connor-McCourt M. (2015). Seminars in Cancer Biology. Semin. Cancer Biol..

[85] Finley S.D., Chu L.-H., Popel A.S. (2014). Computational systems biology approaches to anti-angiogenic cancer therapeutics. Drug Discov. Today.

[86] Qutub A.A. (2013). Systems approaches for synthetic biology: A pathwaytoward mammalian design. Front. Physiol..

[87] Lin Y.C., Huang D.Y., Chu C.L., Lin Y.L., Lin W.W. (2013). The Tyrosine Kinase Syk Differentially Regulates Toll-like Receptor Signaling Downstream of the Adaptor Molecules TRAF6 and TRAF3. Sci. Signal..

[88] Lillemeier B.F. How membrane structures control T cell signaling. 2012, 3, 291. 3.

[89] Zhang Y., Du Y., Le W., Wang K., Kieffer N., Zhang J. (2011). Redox Control of the Survival of Healthy and Diseased Cells. Antioxid. Redox Signal..

[90] Breinig M., Klein F.A., Huber W., Boutros M. (2015). A chemical-genetic interaction map of small molecules using high-throughput imaging in cancer cells. Mol. Syst. Biol..

[91] Reinartz S., Finkernagel F., Adhikary T., Rohnalter V., Schumann T., Schober Y., Nockher W.A., Nist A., Stiewe T., Jansen J.M. (2016). A transcriptome-based global map of signaling pathways in the ovarian cancer microenvironment associated with clinical outcome. Genome Biol..

[92] Speer T., Rohrer L., Blyszczuk P., Shroff R., Kuschnerus K., Kränkel N., Kania G., Zewinger S., Akhmedov A., Shi Y. (2013). Abnormal High-Density Lipoprotein Induces Endothelial Dysfunctionvia Activation of Toll-like Receptor-2. Immunity.

[93] Smyth J.T., Hwang S.-Y., Tomita T., DeHaven W.I., Mercer J.C., Putney J.W. (2010). Activation and regulation of store-operated calcium entry. J. Cell. Mol. Med..

[94] McClean M.N., Mody A., Broach J.R., Ramanathan S. (2007). Cross-talk and decision making in MAP kinase pathways. Nat. Genet..

[95] Stuart R.O., Wachsman W., Berry C.C., Wang-Rodriguez J., Wasserman L., Klacansky I., Masys D., Arden K., Goodison S., McClelland M. (2004). In silico dissection of cell-type-associated patterns of gene expression in prostate cancer. Proc. Natl. Acad. Sci. USA.

[96] Kholodenko B., Yaffe M.B., Kolch W. (2012). Computational Approaches for Analyzing Information Flow in Biological Networks. Sci. Signal..

[97] Kholodenko B.N. (2003). Four-dimensional organization of protein kinase signaling cascades: The roles of diffusion, endocytosis and molecular motors. J. Exp. Biol..

[98] Kholodenko B.N., Demin O.V., Markevich N.I., Kiyatkin A., Moehren G., Hoek J.B. (2004). Signal processing at the Ras circuit: What shapes Ras activation patterns?. Syst. Biol..

[99] Boris N. (2009). Kholodenko Spatially distributed cell signalling. FEBS Lett..

[100] Kholodenko B.N., Kiyatkin A., Bruggeman F.J., Sontag E., Westerhoff H.V., Hoek J.B. (2002). Untangling the wires: A strategy to trace functional interactions in signaling and gene networks. Proc. Natl. Acad. Sci. USA.

[101] Aksamitiene E., Kiyatkin A., Kholodenko B.N. (2012). Cross-talk between mitogenic Ras/MAPK and survival PI3K/Akt pathways: A fine balance. Biochm. Soc. Trans..

[102] Bluthgen N., Bruggeman F.J., Legewie S., Herzel H., Westerhoff H.V., Kholodenko B.N. (2006). Effects of sequestration on signal transduction cascades. FEBS J..

[103] Winstead C.J., Weaver C.T. (2013). Dwelling on T Cell Fate Decisions. Cell.

[104] Nguyen L.K., Matallanas D.G., Romano D., Kholodenko B.N., Kolch W. (2015). Competing to coordinate cell fate decisions: The MST2-Raf-1 signaling device. Cell Cycle.

[105] Reiterer V., Fey D., Kolch W. (2013). Pseudophosphatase STYX modulates cell-fate decisions and cell migration by spatiotemporal regulation of ERK1/2. Proc. Natl. Acad. Sci. USA.

[106] Kumar D., Srikanth R., Ahlfors H., Lahesmaa R., Rao K.V.S. (2007). Capturing cell-fate decisions from the molecular signatures of a receptor-dependent signaling response. Mol. Syst. Biol..

[107] Formosa-Jordan P., Ibañes M. (2014). Competition in Notch Signaling with Cis Enriches Cell Fate Decisions. PLoS ONE.

[108] Li C., Wang J. (2013). Quantifying Cell Fate Decisions for Differentiation and Reprogramming of a Human Stem Cell Network: Landscape and Biological Paths. PLoS Comput. Biol..

[109] Huang W., Cao X., Biase F.H., Yu P., Zhong S. (2014). Time-variant clustering model for understanding cell fate decisions. Proc. Natl. Acad. Sci. USA.

[110] Wang F., Durfee L.A., Huibregtse J.M. (2013). A Cotranslational Ubiquitination Pathway for Quality Control of Misfolded Proteins. Mol. Cell.

[111] Cui Q., Ma Y., Jaramillo M., Bari H., Awan A., Yang S., Zhang S., Liu L., Lu M., O’Connor-McCourt M. (2007). A map of human cancer signaling. Mol. Syst. Biol..

[112] Sanchez-Vega F., Mina M., Armenia J., Chatila W.K., Luna A., La K.C., Dimitriadoy S., Liu D.L., Kantheti H.S., Saghafinia S. (2018). Oncogenic Signaling Pathways in The Cancer Genome Atlas. Cell.

[113] Khosravi M., Poursaleh A., Ghasempour G., Farhad S., Najafi M. (2019). The effects of oxidative stress on the development of atherosclerosis. Biol. Chem..

[114] Tata P.R., Rajagopal J. (2017). Plasticity in the lung: Making and breaking cell identity. Development.

[115] Loukovaara S., Gucciardo E., Repo P., Lohi J., Salven P., Lehti K. (2015). A Case of Abnormal Lymphatic-Like Differentiation and Endothelial Progenitor Cell Activation in Neovascularization Associated with Hemi-Retinal Vein Occlusion. Case Rep. Ophthalmol..

[116] Baylin S.B., Jones P.A. (2011). A decade of exploring the cancer epigenome—Biological and translational implications. Nat. Rev. Cancer.

[117] Michelini R.H., Doedens A.L., Goldrath A.W., Hedrick S.M. (2013). Differentiation of CD8 memory T cells depends on Foxo1. J. Exp. Med..

[118] Chen B., Xue Z., Yang G., Shi B., Yang B., Yan Y., Wang X., Han D., Huang Y., Dong W. (2013). Akt-Signal Integration Is Involved in the Differentiation of Embryonal Carcinoma Cells. PLoS ONE.

[119] Rebhahn J.A., Deng N., Sharma G., Livingstone A.M., Huang S., Mosmann T.R. (2014). An animated landscape representation of CD4 +T-cell differentiation, variability, and plasticity: Insights into the behavior of populations versus cells. Eur. J. Immunol..

[120] Moustakas A., Pardali K., Gaal A., Heldin C.H. (2002). Mechanisms of TGF-β signaling in regulation of cell growth and differentiation. Immunol. Lett..

[121] Ma Y., Adjemian S., Mattarollo S.R., Yamazaki T., Aymeric L., Yang H., Catani J.P.P., Hannani D., Duret H., Steegh K. (2013). Anticancer Chemotherapy-Induced Intratumoral Recruitment and Differentiationof Antigen-Presenting Cells. Immunity.

[122] Quann E.J., Liu X., Altan-Bonnet G., Huse M. (2011). A cascade of protein kinase C isozymes promotes cytoskeletal polarization in T cells. Nat. Publ. Group.

[123] Thomas J.D., Lee T., Suh N.P. (2004). A function-based framework for understanding biological systems. Annu. Rev. Biophys. Biomol. Struct..

[124] Santos S.D.M., Verveer P.J., Bastiaens P.I.H. (2007). Growth factor-induced MAPK network topology shapes Erk response determining PC-12 cell fate. Nat. Cell Biol..

[125] Orton R.J., Adriaens M.E., Gormand A., Sturm O.E., Kolch W., Gilbert D.R. (2009). Computational modelling of cancerous mutations in the EGFR/ERK signalling pathway. BMC Syst. Biol..

[126] Krishnan J., Floros I. (2019). Adaptive information processing of network modules to dynamic and spatial stimuli. BMC Syst. Biol..

[127] Zhang R., Lahens N.F., Ballance H.I., Hughes M.E., Hogenesch J.B. (2014). A circadian gene expression atlas in mammals: Implications for biology and medicine. Proc. Natl. Acad. Sci. USA.

[128] Cheung K.J., Ewald A.J. (2016). A collective route to metastasis: Seeding by tumor cell clusters. Science.

[129] Klein E.A., Cooperberg M.R., Magi-Galluzzi C., Simko J.P., Falzarano S.M., Maddala T., Chan J.M., Li J., Cowan J.E., Tsiatis A.C. (2014). A 17-gene Assay to Predict Prostate Cancer Aggressiveness in the Context of Gleason Grade Heterogeneity, Tumor Multifocality, and Biopsy Undersampling. Eur. Urol..

[130] Kitano H. (2002). Computational systems biology. Nature.

[131] Wang D.Y., Cardelli L., Phillips A., Piterman N., Fisher J. (2009). Computational modeling of the EGFR network elucidates control mechanisms regulating signal dynamics. BMC Syst. Biol..

[132] Klingmüller U. (2012). Heterogeneous kinetics of AKT signaling in individual cells are accounted for by variable protein concentration. Front. Physiol..

[133] Kozer N., Barua D., Orchard S., Nice E.C., Burgess A.W., Hlavacek W.S., Clayton A.H.A. (2013). Exploring higher-order EGFR oligomerisation and phosphorylation--a combined experimental and theoretical approach. Mol. BioSyst..

[134] Di Camillo B., Toffolo G., Cobelli C. (2009). A Gene Network Simulator to Assess Reverse Engineering Algorithms. Ann. N. Y. Acad. Sci..

[135] Stoevesandt O., Kohler K., Wolf S., Andre T., Hummel W., Brock R. (2006). A Network Analysis of Changes in Molecular Interactions in Cellular Signaling. Mol. Cell. Proteom..

[136] Murphy L.O., MacKeigan J.P., Blenis J. (2003). A Network of Immediate Early Gene Products Propagates Subtle Differences in Mitogen-Activated Protein Kinase Signal Amplitude and Duration. Mol. Cell. Biol..

[137] Kim S., Elbaum M. (2013). A Simple Kinetic Model with Explicit Predictions for Nuclear Transport. Biophys. J..

[138] Sontag E., Kiyatkin A., Kholodenko B.N. (2004). Inferring dynamic architecture of cellular networks using time series of gene expression, protein and metabolite data. Bioinformatics.

[139] Vera J., Rath O., Balsa-Canto E., Banga J.R., Kolch W., Wolkenhauer O. (2010). Investigating dynamics of inhibitory and feedback loops in ERK signalling using power-law models. Mol. BioSyst..

[140] Gonzalez J.M., Portillo M.C., Piñeiro-Vidal M. (2015). Latitude-dependent underestimation of microbial extracellular enzyme activity in soils. Int. J. Environ. Sci. Technol..

[141] Varusai T.M., Kolch W., Kholodenko B.N., Nguyen L.K. (2015). Molecular BioSystems. Mol. BioSyst..

[142] Mobasheri A. (2020). Biosensors for the Multiplex Detection of Inflammatory Disease Biomarkers. Biosensors.

[143] Anwer S.T., Mobashir M., Fantoukh O.I., Khan B., Imtiyaz K., Naqvi I.H., Rizvi M.M.A. (2022). Synthesis of Silver Nano Particles Using Myricetin and the In-Vitro Assessment of Anti-Colorectal Cancer Activity: In-Silico Integration. Int. J. Mol. Sci..

[144] Baud V., Karin M. (2009). Is NF-kappaB a good target for cancer therapy? Hopes and pitfalls. Nat. Rev. Drug Disc..

[145] Lin Y., Bai L., Chen W., Xu S. (2010). The NF-kappaB activation pathways, emerging molecular targets for cancer prevention and therapy. Expert Opin. Ther. Targets.

[146] Mobashir M. (2022). The Understanding of the Potential Linkage between COVID-19, Type-2 Diabetes, and Cancer (s) Could Help in Better Drug Targets and Therapeutics. Comb. Chem. High Throughput Screen..

